# The Effects of Promoter Methylation on Downregulation of DAZAP2 in Multiple Myeloma Cell Lines

**DOI:** 10.1371/journal.pone.0040475

**Published:** 2012-07-09

**Authors:** Sai-Qun Luo, Jing-Ping Hu, Qiang Qu, Jiang Li, Wei Ren, Jia-Ming Zhang, Yan Zhong, Wei-Xin Hu

**Affiliations:** Molecular Biology Research Center, Xiangya School of Medicine, Central South University, Changsha, Hunan, People’s Republic of China; Goethe University, Germany

## Abstract

Our previous studies had shown that DAZAP2 was profoundly downregulated in bone marrow mononuclear cells from multiple myeloma patients. In this report, we analyzed epigenetic changes in multiple myeloma cell lines to understand the molecular mechanisms underlying the downregulation of DAZAP2. Four multiple myeloma cell lines, KM3, MM.1S, OPM-2 and ARH-77, were studied. The results of methylation specific PCR (MSP) showed that the promoter of DAZAP2 was methylated for KM3, MM.1S, OPM-2 and unmethylated for ARH-77. The DAZAP2 promoter region was amplified to obtain a series of different length sequences. All of the amplified sequences were inserted to luciferase reporter vector. The constructs were transfected into COS-7 cells and the luciferase activities were measured to search for the core region of DAZAP2 promoter. Two CpG islands were found in DAZAP2 promoter region. The results of luciferase assay showed that CpG island 1 displayed weak transcriptional activity, whereas CpG island 2 exhibited strong transcriptional activity (273 folds) compared to the control. The sequence that covered both CpG islands 1 and 2 showed higher activity (1,734 folds) compared to the control, suggesting that the two islands had synergistic effect on regulating DAZAP2 expression. We also found that M. *Sss* I methylase could inhibit the luciferase activity, whereas demethylation using 5-aza-2′-deoxycytidine treatment rescued the expression of DAZAP2 for multiple myeloma cell lines. These data revealed that methylation of DAZAP2 promoter was involved in downregulation of DAZAP2 in multiple myeloma cells.

## Introduction

Multiple myeloma is a disease with the uncontrolled proliferation and accumulation of malignant plasma cells in the bone marrow. Multiple myeloma cells are characterized by a profound genetic instability resulting in a complex set of numerical and structural chromosomal abnormalities. Pathogenesis of multiple myeloma cells was a multistep process in which plasma cell suffered series of molecular and cellular changes [1], [2].Constitutive genetic alterations of multiple myeloma cells are important determinants of the biological behavior of multiple myeloma cells in their local microenvironment. The interaction of multiple myeloma cells with BMSCs and bone marrow accessory cells upregulated transcripts for cytokines such as IL-6 [3], [4], the anti-apoptotic protein MCL1 [5]; HGF and insulin-like growth factors (IGFs) [6]; and heat shock proteins (HSPs), which regulate the conformation and function of proliferative and anti-apoptotic proteins [7]. This increased autocrine production of cytokines, along with paracrine cytokine production from the BMSCs, stimulates proliferative and anti-apoptotic signaling cascades in multiple myeloma cells. Bone destruction in multiple myeloma is mediated by MIP1α. MIP1α is a potent inducer of osteoclast formation independent of RANKL, and promotes both RANKL-stimulated and IL6-stimulated osteoclast formation [8]. Dickkopf 1 (DKK1) and IL-3 may contribute to the inhibitory effects of multiple myeloma cells on osteoblast differentiation. DKK1 inhibits the canonical Wnt pathway, which mediates the differentiation of osteoblast progenitor cells, DKK1 expression of multiple myeloma cells could inhibit osteoblastogenesis. Increased DKK1 levels in bone marrow plasma and peripheral blood from patients with multiple myeloma are associated with focal bone lesions [9], [10]. This imbalance between bone formation and resorption results in osteolytic lesions, which is a hallmark of multiple myeloma.

The epigenetic alterations are among the earliest molecular abnormalities to occur during tumorigenesis. A variety of genetic changes had been detected in multiple myeloma, including alterations in methylation of p16 gene, a frequent epigenetic event in multiple myeloma patients [11]. CpG island methylation of DKK1 promoter is correlated with in several MM cell lines and in MM cells from advanced MM patients. Demethylation of the DKK1 promoter restores DKK1 expression, which results in inhibition of β-catenin/TCF-mediated gene transcription in MM lines [12]. The chromosomal region at 13q14.1-14.3 is a hotspot of deleting abnormalities, and it appears that some multiple myeloma-specific tumor suppressor genes may reside in this region [13]. Some of immunoglobulin heavy-chain (IgH) translocations were recurrent and had characterized the genomic breakpoints of seven t(4;14) translocations from multiple myeloma patients [14]. The rearrangements of the IgH locus at 14q32 were found in the majority cases of multiple myeloma. p18 was also found to be frequently deleted in multiple myeloma patients [15]. All these abnormalities represented downregulation of tumor suppressor genes.

**Table 1:** Primers for amplification of DAZAP2 promoter.

Sequence name	Primers	Location	Length
S1	P1∶ 5′-CGA GCT CGC TGC CTA GGC TGG TTC TGA AC-3′P11∶ 5′-GGA AGA TCT CAG GAA CTC TCC TGG GCC TAC-3′	−1980→−1677	304 bp
S2	P1∶ 5′-CGA GCT CGC TGC CTA GGC TGG TTC TGA AC-3′P12∶ 5′-GGA AGA TCT CGA TTC TCC TGC CTC AGC CT-3′	−1980→−1506	475 bp
S3	P1∶ 5′-CGA GCT CGC TGC CTA GGC TGG TTC TGA AC-3′P13∶ 5′-GGA AGA TCT CTC GAA CTC CTG ACC TCA GGT G-3′	−1980→−1281	700 bp
S4	P1∶ 5′-CGA GCT CGC TGC CTA GGC TGG TTC TGA AC-3′P14∶ 5′-GGA AGA TCT GTT CAC TGC AAC CTC CGC CTC-3′	−1980→−1134	847 bp
S5	P1∶ 5′-CGA GCT CGC TGC CTA GGC TGG TTC TGA AC-3′P15∶ 5′-GGA AGA TCT GAC GGA GTC TTG CTC GCT TGC-3′	−1980→−831	1153 bp
S6	P1∶ 5′-CGA GCT CGC TGC CTA GGC TGG TTC TGA AC-3′P16∶ 5′-GGA AGA TCT GTC GCA CTG CCT GAA ATC GG-3′	−1980→−390	1591 bp
S7	P1∶ 5′-CGA GCT CGC TGC CTA GGC TGG TTC TGA AC-3′P17∶ 5′-GGA AGA TCT TAC CGG TGT CCC TCA GCT-3′	−1980→−201	1780 bp
S8	P1∶ 5′-CGA GCT CGC TGC CTA GGC TGG TTC TGA AC-3′P2∶ 5′-GGA AGA TCT GCC TTT GCT GTT CAT GGT GG-3′	−1980→+58	2038 bp
S9	P21∶ 5′-CGA GCT CTT CTC CAG CTG AGG GAC ACC G-3′P2∶ 5′-GGA AGA TCT GCC TTT GCT GTT CAT GGT GG-3′	−224→+58	282 bp
S10	P22∶ 5′-CGA GCT CGC CGA TTT CAG GCA GTG CG-3′P2∶ 5′-GGA AGA TCT GCC TTT GCT GTT CAT GGT GG-3′	−410→+58	468 bp
S11	P23∶ 5′-CGA GCT CGC AAG CGA GCA AGA CTC CGT C-3′P2∶ 5′-GGA AGA TCT GCC TTT GCT GTT CAT GGT GG-3′	−848→+58	906 bp
S12	P24∶ 5′-CGA GCT CGA GGC GGA GGT TGC AGT GAA C-3′P2∶ 5′-GGA AGA TCT GCC TTT GCT GTT CAT GGT GG-3′	−1154→+58	1212 bp
S13	P25∶ 5′-CGA GCT CTG GAT CAC CTG AGG TCA GGA G-3′P2∶ 5′-GGA AGA TCT GCC TTT GCT GTT CAT GGT GG-3′	−1307→+58	1365 bp
S14	P26∶ 5′-CGA GCT CGA GGC TGA GGC AGG AGA ATC G-3′P2∶ 5′-GGA AGA TCT GCC TTT GCT GTT CAT GGT GG-3′	−1526→+58	1584 bp
S15	P27∶ 5′-CGA GCT CGT AGG CCC AGG AGA GTT CCT G-3′P2∶ 5′-GGA AGA TCT GCC TTT GCT GTT CAT GGT GG-3′	−1697→+58	1755 bp

Growth factors and cytokines may also be involved in the regulation of plasma cell proliferation and tumor progression. For example, IL-1β, IL-6, IL-10, IFN-α, IGF-1 and TGF-γ promote proliferation of multiple myeloma cells, whereas IFN-β1 and APO-1/FAS inhibit the growth of multiple myeloma cells [16]–[19]. The cyclin D1 promoter was hypomethylated and hyperacetylated in expressing cell lines and patient samples. RNA polymerase II bound at IgH regulatory sequences can activate the cyclin D1 promoter by either long-range polymerase transferring or tracking [20]. RAS mutations often provide a genetic marker, if not a causal event, in the evolution of MGUS (monoclonal gammopathy of undetermined significance) to multiple myeloma and play a role in the transition from intramedullary to extramedullary tumor [21]. Cell adhesion molecules and cell surface antigens may further contribute to the localization of myeloma cells in the bone marrow [22].

DAZAP2 (deleted in azoospermia associated protein 2) had originally been identified as an interacting protein of germ-cell-specific RNA-binding proteins DAZ (deleted in azoospermia) [23].The evolutionary conserved DAZAP2 protein functions as a TCF-4 interacting partner. The knockdown of DAZAP2 could not only reduce the activity of Wnt-signaling as measured by Tcf/−catenin reporters but also additionally alter the expression of Wnt-signaling target genes [24]. DAZAP2 modulates the affinity of TCF-4 for its DNA-recognition motif in chromatin immunoprecipitation studies. Loss of DAZAP2 in embryos resulted in diminished expression of *hoxb9* with a concurrent increase in the anterior marker *otx2*. DAZAP2 is required for FGF dependent posterior patterning. In contrast to FGF activity, DAZAP2 induction of *hoxb9* is not blocked by loss of canonical Wnt-signaling, and increasing DAZAP2 level alters neural patterning and induces posterior neural markers [25].

Our previous studies showed that DAZAP2 was the most profoundly downregulated gene in bone marrow mononuclear cells from multiple myeloma patients [26], [27]. The results implicated a role for DAZAP2 as a potential tumor suppressor involved in the origin and development of multiple myeloma. However, the mechanism of its downregulation is so far unknown.

**Table 2:** Primers for amplification of CpG islands.

Name	Primers	Location	Length
CpG1-f	5′-CTA GCT AGC GAT TTC AGG CAG TGC GAC A-3′	−175→−408	251 bp
CpG1-r	5′ -CCG CTC GAG CTC GCT CGT TTC TGT CCT C-3′		
CpG2-f	5′-CTA GCT AGC GGA GGA CAG AAA CGA GC-3′	−194→+51	262 bp
CpG2-r	5′-CCG CTC GAG CTG TTC ATG GTG GTT GC-3′		

## Materials and Methods

### Cell Lines

The human multiple myeloma cell line ARH-77, OPM-2, MM.1S and African monkey kidney cell line COS7 were from the American Type Culture Collection (ATCC; Manassas, VA). The human multiple myeloma cell line KM3 was from the Cell Center of Shanghai Chuan-Xiang Biotechnology (Shanghai, China). The cells were cultured in medium RPMI 1640 or DMEM (Gibco) supplemented with 10% heat-inactivated fetal bovine serum (FBS; Gibco), 100 kU/L benzylpenicillin, and 100 mg/L streptomycin at 37°C in a humidified incubator containing 5% CO_2_.

### Reagents, Polymerases and Restriction Endonucleases, Reportor Gene Vectors and Primers

*Taq* DNA polymerases, restriction endonuclease such as *Xho* I, *Nhe* I, and T4 DNA ligase were from TaKaRa (Dalian, China). CpG methylation enzyme *Sss* I, AMV Reverse transcriptase and restriction endonuclease *Bst*U I were from the New BioLabs. Agarose gel DNA purification kit was from Ambiogen. Plasmids purification mini kit, DNA Wizard clean-up system, the pGL2 Luciferase Reporter Vectors, Luciferase Assay System, β-galactosidase immuosorbent were from Promega. Lipofectamine™ LTX regent was from Invitrogen. 5-aza-2′-deoxycytidine was from Sigma-Aldrich.

pGL2 vector series containing luciferase reporter gene include of pGL2-Basic plasmid (lacking of promoter and enhancer) as negative control and pGL2-Control plasmid (including SV40 promoter and enhancer) as positive control. pSV-β-gal was used as an internal control to normalize the differences in transfection efficiency.

All primers were synthesised by Invitrogen.

### Construction of Recombinant Plasmids

Genomic DNA was extracted from multiple myeloma cell lines. PCR amplification system were as follows: 1–2 µl of genomic DNA (approximately 100 ng),5 µl of 10×Buffer,4 µl of dNTP (2.5 mM), 1 µl of sense (F)/antisense (R) primers (10 µM) (Table 1 and 2), 2 U *Taq* DNA polymerase, and distilled H_2_O was added up to 50 µl. PCR amplification conditions were as follows: hot-started at 94°C for 3 min,30 cycles of 94°C for 30 s, 60°C for 30 s and 72°C for 30 s, followed by one cycle of 72°C for 5 min. PCR products were purified by agarose gel DNA purification kit. The amplifying DNA fragments and pGL2 vector were digested with *Xho* I and *Nhe* I respectively, and ligated in the presence of T4 DNA ligase.

### Analysis of DAZAP2 Expression Level in Multiple Myeloma Cell Lines

0.5 µg of total RNA extracted from above-mentioned cell lines were mixed with 0.5 µg of oligo(dT)_15_, then heated for 5 min at 65°C and chilled on ice for 5 min. The reaction system included 4 µl of 5×RT buffer, 20 U RNasin, 4 µl of dNTP (2.5 mM), 10 U AMV Reverse transcriptase (RT) and DEPC treated water was added to 20 µl. The mixture was incubated for 60 min at 42°C. Then the reaction was terminated by heating for 5 min at 95°C.

Fifty µl PCR reaction mixtures included 1.5 mM MgCl_2_, 200 µM dNTPs, 1 µl of 10 µM specific primers, 2.5 U *Taq* DNA polymerase, 5 µl of cDNA template and distilled water was added to 50 µl. Reaction condition was as follows: hot-started for 2 min at 94°C,32 cycles of 94°C for 30 s, 56°C for 30 s and 72°C for 30 s, followed by one cycle of 72°C for 5 min. β-actin was used for normalization of the relative expression level of mRNA, and PCR reaction was stopped before plateau phase. Primers for DAZAP2 are 5′-ACA GCC AAC CTA CCC TGT GCA-3′ (forward) and 5′-CAT GAC TGC AAG CTG AGC AGC-3′ (reverse) Primers for β-actin are 5′-TGA CGG TCA GGT CAT CAT TAT CGG CAA TGA-3′ (forward) and 5′-TTG ATC TTC ATG GTG ATA GGA GCG AGG GCA-3′ (reverse).

**Figure 1:**
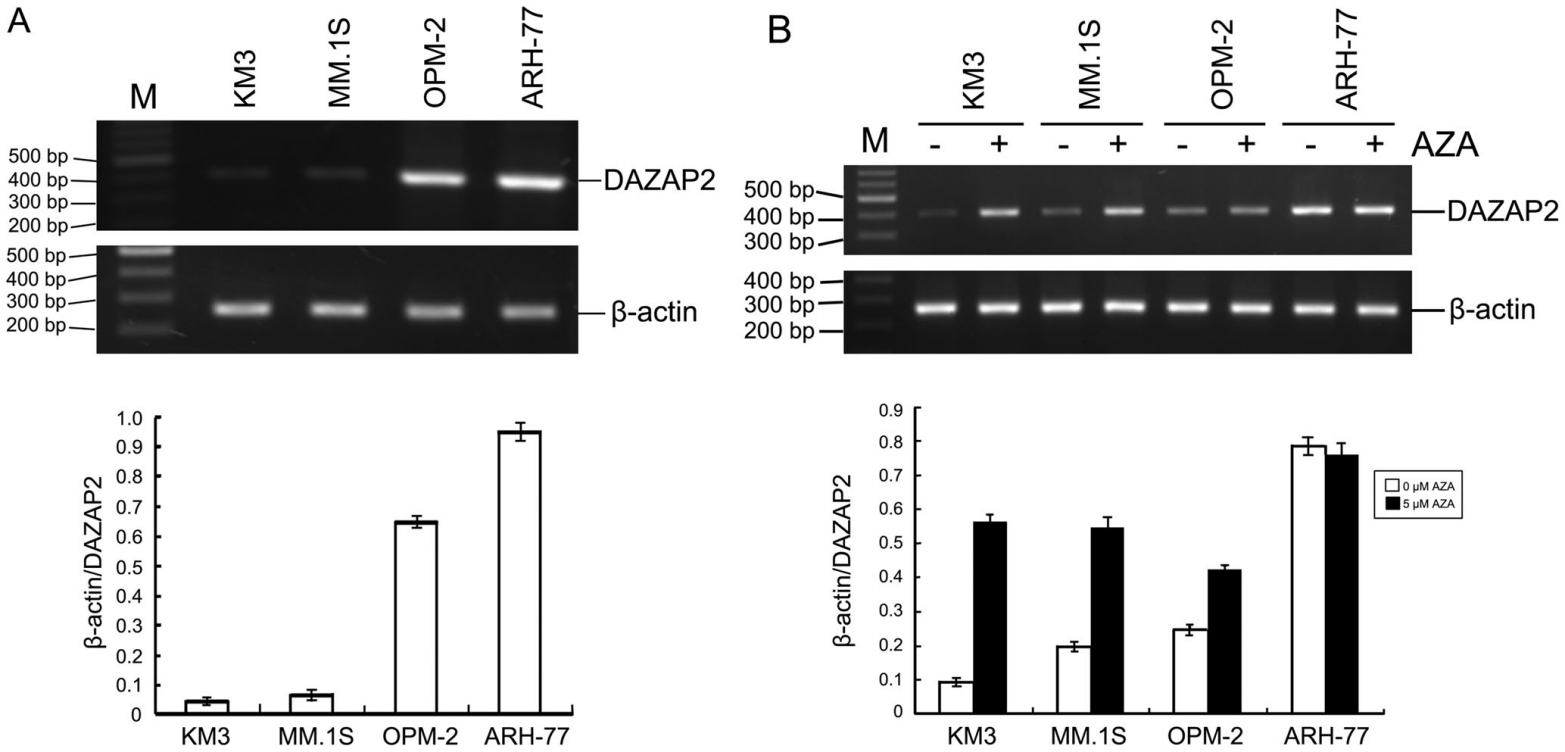
DAZAP2 gene expression in multiple myeloma cell lines KM3, MM.1S, OPM-2, and ARH-77. (A) DAZAP2 gene expression in normal condition. (B) DAZAP2 gene expression after treatment with 5-aza-2′-deoxycytidine. The total RNA was isolated from multiple myeloma cells and RT-PCR was performed with the specific primers to detect the gene expression level of DAZAP2. The PCR products were ran on 1.5% agarose gel. β-actin was used for normalization and verification of sample loading. The length of amplification products for DAZAP2 is 400 bp and for β-actin is 260 bp. M:100 bp DNA ladder plus marker; AZA: 5-aza-2′-deoxycytidine; (−): without treatment of 5-aza-2′-deoxycytidine as control; (+): with treatment of 5-aza-2′-deoxycytidine. Each value represents mean ± S.D. of three independent experiments.

**Figure 2:**
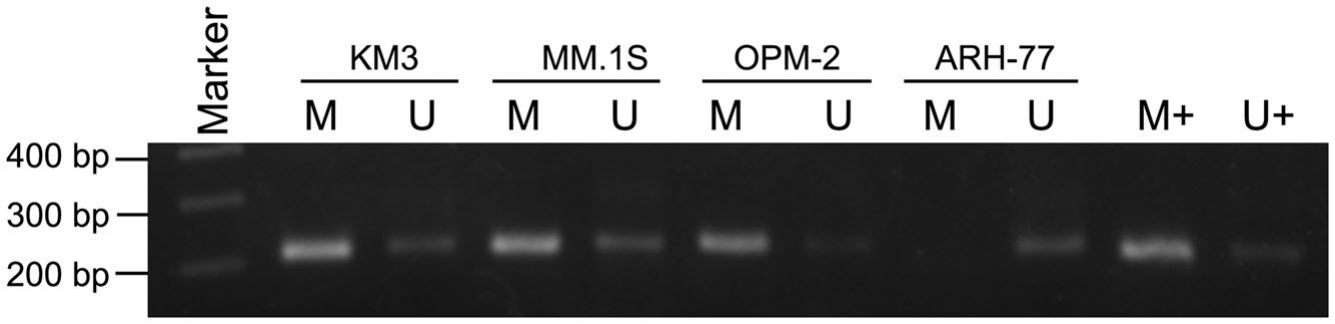
MSP analysis of DAZAP2 promoter methylation status in multiple myeloma cell lines KM3, MM.1S, OPM-2, and ARH-77. MSP was performed with the specific primers to amplify the target sequence and a fragment with length of 214 bp was obtained. M: 100 bp DNA ladder plus marker; U: unmethylated sequence; M: methylated sequence; M+: positive methylation control; U+: negative methylation control.

### Multiple Myeloma Cell Lines were Treated with 5-aza-2′-deoxycytidine

5-aza-2′-deoxycytidine was dissolved in 50% acetic acid before use. Multiple myeloma cell lines KM3, MM.1S, OPM-2, and ARH-77(1×10^5^/ml)were cultured in the medium RPMI 1640 with 10% calf serum for 24 h. The cells were treated with 5 µM 5-aza-2′-deoxycytidine or with solvent as control (0 µM 5-aza-2′-deoxycytidine). The final concentration of acetic acid in the medium was 0.025%. Cells were washed with PBS twice, fresh medium and 5-aza-2′-deoxycytidine were added for every 24 hrs. After triple treatment, cells were collected and total RNA was isolated. RT-PCR was performed to detect the gene expression of DAZAP2 in KM3, MM.1S, OPM-2, and ARH-77 cells.

**Figure 3:**
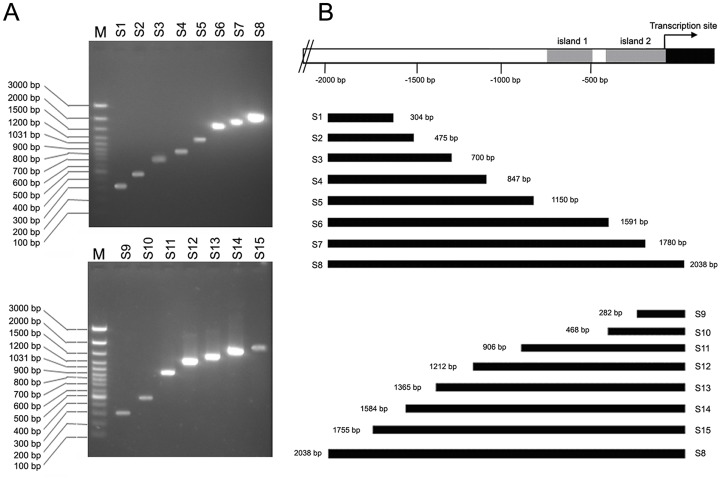
Amplification of DAZAP2 promoter region. DAZAP2 promoter region is located upstream of DAZAP2 transcription site. The PCR products were a series of different length sequences. S1∼S15 are the names of DNA fragments corresponding to specific primers in Table 1. (A) the PCR products running on agarose gel; (B) the schematics of amplified DNA fragments in the location of DAZAP2 promoter region.

**Figure 4:**
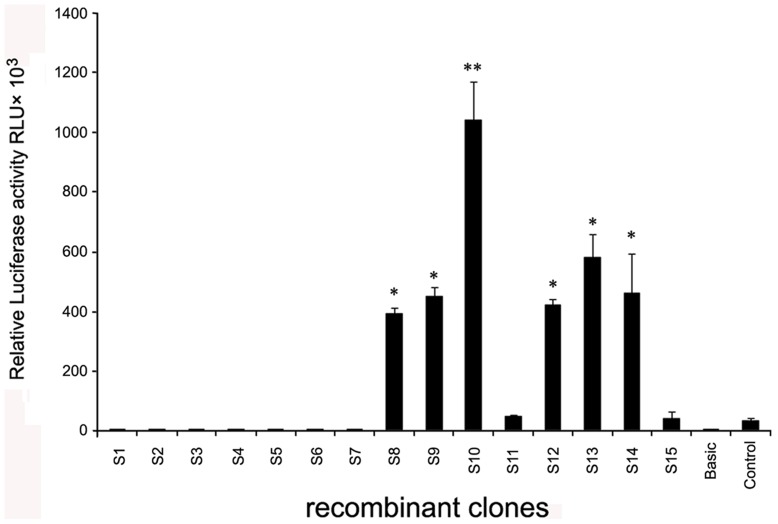
The activity analysis of promoter region upstream of DAZAP2 gene. The amplified DNA fragments in DAZAP2 promoter region were inserted to luciferase reporter gene vector pGL2-Basic and luciferase activity was detected. The empty vector pGL2-Basic was used as negative control. The vector pGL2-Control, containing SV40 promoter and enhancer, can express luciferase with high efficiency and was used as positive control. Correction value = (S_RLU_-B_RLU_)/(S_β-G_-B_β-G_); S: recombinant clone sample; B: negative control; RLU (relative light unit): luciferase activity; β-G: β- galactosidase activity. Results are the Mean ± S.D of 3 independent experiments and asterisk symbol indicates P<0.05(*) or P<0.01(**) compared with the control group.

### Analysis of Methylation Specific PCR (MSP)

Fifty µl of genomic DNA (2 µg) was denatured in 5.5 µl of fresh prepared NaOH (3 M) for 15 min at room temperature. The denatured DNA was mixed with freshly prepared 30 µl of 10 mM hydroquinone and 520 µl of 3 M sodium bisulfate. After incubated for 16 h at 50°C in the dark, the treated DNA was purified using DNA Wizard clean-up system and dissolved in 50 µl water. 33 µl of 10 M ammonium acetate and 270 µl anhydrous alcohol were added and then incubated at −80°C so as to precipitate DNA. The solution was centrifuged for 30 min and washed with ice-cold 70% ethanol. The pellet was resuspended in 20 µl distilled water and stored at −20°C.

Two µl of bisulfite-treated genomic DNA was mixed with 2.5 µl of 10×buffer, 2 µl of 2.5 mM dNTPs,0.5 µl of 10 µM primers M1/M2 or U1/U2, 0.3 µl of 1 U/µl *Taq* DNA polymerase and distilled water was added to 25 µl. Amplification conditions were as follows: hot-started at 94°C for 2 min, 40 cycles of 94°C for 30 s, 53°C for 30 s and 72°C for 30 s, followed by one cycle of 72°C for 5 min. 5 µl of PCR products were run on a 2% agarose gel stained with ethidium bromide to observe the results. Primers (M1/M2) for methylation analysis are 5′-TTT TTT TTA GTT GAG GGA TAT CGG-3′ and 5′-CTT CCT ATT CGA AAC TAC TTC CGT A-3′. Primers (U1/U2) for unmethylation analysis are 5′-TTT TTT TTA GTT GAG GGA TAT TGG-3′ and 5′-CTT CCT ATT CAA AAC TAC TTC CAT A-3′.

**Figure 5:**
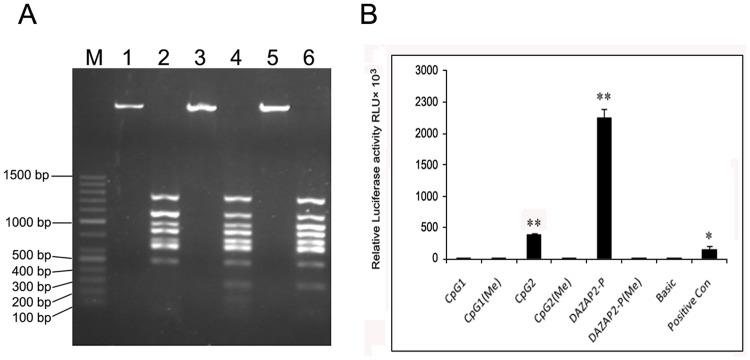
Effects of methylated DAZAP2 promoter region on transcription activity. (A) Methylated and unmethylated recombinant plasmids were digested by *Bst*U I and run on agarose gel. M: 100 bp ladder plus marker; 1, 3, 5: methylated pGL2-DAZAP2/CpG1, CpG2 and DAZAP2; 2, 4, 6: unmethylated pGL2-DAZAP2/CpG1, CpG2 and DAZAP2. (B) Recombinant reporter plasmids bearing methylated DNA fragment were constructed (see materials and methods). COS-7 cells were transfected with recombinant reporter plasmids bearing methylated and unmethylated DNA fragment. pSV-β-gal plasmid was used as an internal control reporter to normalize the differences in transfection efficiency. pGL2-Control vector as positive control and pGL2-Basic vector as negative control were used respectively. Relative luciferase activity was measured in triplicates. Results are the Mean ± S.D of 3 independent experiments and asterisk symbol indicates P<0.05(*) or P<0.01(**) compared with the control group).

### Methylation of CpG Island Fragment and Construction of pGL2–DAZAP2/CpG1(Me), pGL2–DAZAP2/CpG2(Me), pGL2–DAZAP2(Me) Vectors

pGL2–DAZAP2/CpG1, pGL2–DAZAP2/CpG2 and pGL2–DAZAP2 were digested by *Xho I* and run on a 0.8% agarose gel with ethidium bromide, then purified by agarose gel DNA purification kit. Methylation assay was incubated for 4 hrs at 37°C as follows: 10 µl of 10×NE buffer II, 10 µl of 10× SAM, 5 µg of digested fragment, 0.5 µl of *Sss* I methylation enzyme and water was added to 100 µl. Ten µl of methylation mixture was added 0.5 U restriction endonuclease *Bst*U I, then incubated for 1 hr at 37°C. Methylated reaction efficiency was observed by electrophoresis on 0.8% agarose gel.

DNA fragments methylated by *Sss* I were digested by *Nhe* I and inserted into linear pGL2-Basic vector which was digested by *Nhe* I/*Xho* I. Ligation products were purified by agarose gel DNA purification kit. The new recombinant plasmids with methylated fragments were named as pGL2-DAZAP2/CpG1(Me), pGL2-DAZAP2/CpG2(Me) and pGL2-DAZAP2(Me).

### Transfection

COS7 cells in exponential growth phase were rinsed twice with PBS, treated with 0.25% trypsin and added to the DMEM media with 10% fetal bovine serum (FBS). The cells were plated on 24-well plates at density of 0.4×10^5^/well in 500 µl DMEM with 10% FBS. Twenty-four hours later, cells of 80% attachment were transfected with recombinant plasmid. Transfection reagent was diluted with serum and antibiotics-free DMEM medium, and then mixed with 0.4 µg plasmid DNA. The mixture above was incubated at room temperature for 20 min. After moving medium in the plates, cells were washed with PBS and then added into 24-well plates. After 6–8 hrs, medium was changed by fresh DMEM with 10% FBS.

### Reporter Gene Activity Assay

After 24–48 hrs of COS7 cells transfection, cell lysate was prepared. DMEM was removed from plates and rinsed with PBS twice. Two hundred µl 1×RLB (reporter lysis buffer) was added per well and the plates were rocked for several times to ensure complete coverage of the cells. Cells were scraped from plates and transferred to 1.5 ml Eppendorf tube on ice. Vortex for 10 s and centrifugation at 12,000×g for 2 min were performed at 4°C. Supernatant was transferred to a new tube and stored at −80°C before use.

Luminometer was programmed to perform a 2-second measurement delay followed by a 10-second measurement read for luciferase activity. One hundred µl of the Luciferase Assay Reagent and 20 µl cell lysate were added into luminometer tube per sample and mixed by pipetting 2–3 times. Tube was placed in the luminometer and results were recorded. The assay was performed in triplicate, and each experiment was repeated at least three times. β- galactosidase activity assay was performed to correct the the value of luciferase activity. Ten µl of cell lysate and 140 µl of 1×RLB were mixed with 150 µl of 2×buffer (ice-cold) and pipetted 2–3 times. Mixture was incubated for 30 min at 37°C. Five hundred µl of 1 M sodium carbonate was added to the tube to terminate the reaction.

### Statistical Analysis

All data were expressed as mean ± standard deviation (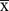
± SD). Statistical analyses were performed with One-way ANOVA for significance using SPSS11.5 software. P value of less than 0.05 (*P*<0.05) indicated a significant difference between groups.

## Results

### DAZAP2 Expression in Multiple Myeloma Cell Lines

Total RNA was extracted from multiple myeloma cell lines KM3, MM.1S, OPM-2 and ARH-77. RT-PCR analysis was performed and the results showed that all cell lines had the band of DAZAP2 (400 bp). The expression level of DAZAP2 in KM3, MM.1S, OPM-2 was lower than that in ARH-77 (Fig.1A).

### Effect of 5-aza-2′-deoxycytidine on DAZAP2 Gene Expression in Multiple Myeloma Cell Lines

The above-mentioned multiple myeloma cell lines(1×10^5^/ml)were respectively seeded in plates, cultured in RPMI 1640 medium with 10% FBS. Twenty four hours later, cells were treated with demethylation reagent 5-aza-2′-deoxycytidine at concentration of 0 and 5 µM. The medium was changed and 5-aza-2′-deoxycytidine was added every 24 hrs for 3 times. Cells were collected and total cellular RNA was isolated 24 hrs later at 3rd treatment. RT-PCR was performed so as to evaluate DAZAP2 gene expression level in KM3, MM.1S, OPM-2 and ARH-77 cells. The results showed that the expression level of DAZAP2 in KM3, MM.1S, OPM-2 with treatment of 5-aza-2′-deoxycytidine was much more than that without treatment of 5-aza-2′-deoxycytidine (Fig. 1B). However, there was no change of DAZAP2 expression level in ARH-77 cells after treatment of 5-aza-2′-deoxycytidine.

### MSP Analysis of DAZAP2 Promoter

Genomic DNA was extracted from multiple myeloma cell lines. MSP analysis was performed so as to analyze DAZAP2 promoter methylation status in KM3, MM.1S, OPM-2 and ARH-77 cells and results showed that both methylated and unmethylated product could be detected in KM3, MM.1S, OPM-2 cells, while in ARH-77 cells only unmethylated product could be detected. Results indicated that DAZAP2 promoter was partially methylated at least in KM3, MM.1S, OPM-2 cells, while unmethylated in ARH-77 (Fig.2). This could explain that why the expression level of DAZAP2 in KM3, MM.1S, OPM-2 was lower than that in ARH-77.

### Amplification of DAZAP2 Promoter Region and Activity Analysis

The DAZAP2 promoter region is located in the upstream of DAZAP2 transcription site and is a GC-rich region (GenBank accession number: JQ425692). The primers were designed to amplified this region (Table 1) and the PCR products were a series of different length sequences (Fig. 3). Bioinformatic analyses showed that there were 2 CpG islands (island 1 and island 2) in DAZAP2 promoter region. To search for the core region of DAZAP2 promoter, all of the amplified sequences were inserted to luciferase reporter gene vector pGL2-Basic. After transfection of COS7 with recombinant plasmids, luciferase activity was detected. Recombinant plasmid containing S10 sequence displayed most active than others (Fig. 4), while island 2 is in it. These results were consistent with bioinformatic analysis of DAZAP2.

### Effect of Methylation-treated DAZAP2 Promoter on Transcription Activity

Recombinant plasmids were linearized by *Xho* I digestion and methylated by *Sss* I. Methylation efficiency was assessed by *Bst*U I digesting methylated linear fragment. Because *Bst*U I hardly digested methylated cytosine (C^m^) sequence, methylated linear fragment was integral. However unmethylated or incompletely methylated fragments were digested into fragments of varying sizes by *Bst*U I (Fig. 5A).

To explore the effects of methylated DAZAP2 promoter region on transcription activity, recombinant reporter plasmids bearing methylated DNA fragment were constructed and COS7 cells were transfected. Compared to pGL2-Basic, luciferase activity of pGL2- DAZAP2/CpG1 was only 7.7 fold (P>0.05) while pGL2-DAZAP2/CpG2 was 273 fold (**P<0.01). Data showed that CpG island 2 exerted an extensive activity, however CpG island 1 displayed inconspicuous activity. Relative luciferase activity of pGL2-DAZAP2 (which covered island 1 and island 2) was 1734 fold of pGL2-Basic (**P<0.01). It was indicated that two CpG islands coalition exerted stronger promoter activity, suggesting that the two islands had synergistic effect on regulating DAZAP2 expression. Relative luciferase activity of pGL2-DAZAP2/CpG1(Me), pGL2-DAZAP2/CpG2(Me) and pGL2-DAZAP2(Me) had no obvious difference compared to pGL2-Basic (P>0.05), which indicated that promoter activity was apparently inhibited attribute to methylated CpG loci blocked the expression of luciferase gene (Fig. 5B).

## Discussion

To further understand the molecular mechanism underlying this deregulation, we analyzed epigenetic changes associated with DAZAP2 in multiple myeloma, given that aberrant methylation of promoter contributes to tumorigenesis by inducing transcriptional suppression and tumor suppressor inactivation.

Most malignant features of cancer cells are triggered by activated oncogenes and the loss of tumor suppressors due to mutation or epigenetic inactivation. We previously reported that DAZAP2 was downregulated in newly diagnosed multiple myeloma and this may influence the growth and survival of multiple myeloma cell [26], [27]. The present study was conducted to evaluate the mechanism of DAZAP2 downregulation and determine the methylation status of its promoter in multiple myeloma cells. Two islands with rich GC box in the promoter of DAZAP2 gene were identified. These data indicated that DAZAP2 promoter was hypermethylated and suppressed the expression of DAZAP2 in multiple myeloma cells. We further correlated DAZAP2 expression with normal plasma cells and malignant myeloma cells, as well as the molecular subtypes which the dataset includes 8 genetic subtypes (MY, PR, LB, MS, HP, CD-1, CD-2, and MF) from 351 newly diagnosed myeloma cases [28]. Interestingly, we extended and confirmed our previous discovery that DAZAP2 was significantly downregulated in multiple myeloma cells by using a large uniform dataset (P = 0.004). The low expression of DAZAP2 was especially significant in the subgroups of MY, PR, LB, HP, and CD-1 [28]. This study warrants further investigation of DAZAP2 and its potential role in multiple myeloma. It is indicated that the promoter hypermethylation played a major role in downregulation of DAZAP2 gene expression in multiple myeloma.

DNA methyltransferases (DNMTs) play an important role in maintaining DNA methylation. Abnormal expression of DNMTs and their isoforms have been found in many types of cancer and different DNMT domains are responsible for targeting DNA methylation to specific regions of the genome [29]. An interest in the status of gene methylation has grown in tumor studies in recent years [30], [31]. In 1983, a relationship between the incidence of malignancy and the abnormality of DNA methylation status was confirmed. Some genes in tumor cells exhibit low methylation level, whereas others exhibit high methylation level [32]. Aberrant methylation of genes is an important mechanism for inactivating the genes involved in tumorigenesis [33], [34].

Research has indicated that aberrant gene methylation is an important mechanism of deregulation in tumor cells and methylation of pro-apoptotic genes may accelerate the malignant transformation of tumors. Several genes involved in cell signaling were aberrantly methylated in AML, and the p73 methylation rate was 13.3% [35]. The hypermethylation of the promoter plays a critical role in the inactivation of cancer-associated genes, and some methylated cancer-associated genes may become an independent prognostic factor for their correlated disease. The methylation status may be becoming a new biological marker and risk factor for ALL [36].

Methylation is one kind of reversible genetic variation. Demethylation can be used to reactivate the expression of some important tumor suppressor genes in tumors or precancerous lesions, therefore, it may play a role in tumor therapy or prevention. Demethylation reagents, such as 5-aza-2′-deoxycytidine, are capable of reactivating methylated genes. *GSTP1* DNA methylation and protein expression status is correlated with 5-aza-2′-deoxycytidine treatment response in prostate cancer cells [37]. A DNA methylation inhibitor demethylated p73 and activated p21 protein to promote AML cell apoptosis [38]. Recently, 5-aza-2′-deoxycytidine had been used in clinical trials, especially in hematological malignancies. The demethylating drug has been shown to be effective in treating patients with myelodysplastic syndrome (MDS) and blast crisis of chronic myelogenous leukemia (CML-BL) [39], [40]. Recent studies have indicated that the therapeutic effect of 5-aza-2′-deoxycytidine is associated with not only demethylation but also DNA covalent conjugation. Therefore, 5-aza-2′-deoxycytidine induced structural changes in chromatin may be caused by histone acetylation and methylation [41].

The present work provided the direct evidence between methylation of DAZAP2 promoter and downregulation of DAZAP2 in multiple myeloma cell lines. DAZAP2 promoter methylation might be involved in tumorigenesis of multiple myeloma and this would be helpful for the diagnosis and treatment of multiple myeloma patients.
